# Supplementary material for: The effect of genome graph expressiveness on the discrepancy between genome graph distance and string set distance
Source: Bioinformatics. 2022 Jun 27;38(Suppl 1):i404–12. doi: 10.1093/bioinformatics/btac264 (PMC9235494; doi:10.1093/bioinformatics/btac264)
Supplement: btac264_Supplementary_Data [file btac264_supplementary_data.pdf]

Supplementary Material for
The Effect of Genome Graph Expressiveness on the Discrepancy Between Genome Graph Distance and String Set Distance

Yutong Qiu[1] and Carl Kingsford[1*]

[1]Compuational Biology Department, Carnegie Mellon University,

Pittsburgh, PA, United States 15232

S1 Procedures to generate data sets

	Group 1	Group 2	Group 3	Group 4	Group 5
TRB_V	5	34	63	92	121
TRB_J	5	8	11	14	15
TRB_D	3	3	3	3	3

Table S1: The number of unique V, J and D gene sequences in each reference gene group.

S1.1 Synthetic Sets of T-cell Receptor Sequences

We construct five reference gene groups by sampling reference sequences obtained from the IMGT database [2] that represent varied diversities of V, D, J gene repertoires. The number of sequences in each group is shown in Table S1. We construct five TCR sequence groups, and each group of TCR sequences are constructed using genes from one of the five reference gene groups. To generate each TCR sequence, we randomly select a V, D and J gene from corresponding gene group, and randomly introduce $m \in \{1, 3, 5, 8, 10\}$ single-nucleotide mutations to each sequence at random locations. This step is to simulate recombination and occurrences of junction single nucleotide polymorphisms (SNPs). 500 sequences are generated in each TCR sequence group.

We construct 50 immune repertoires in five groups. Each repertoire group are constructed using simulated TCR sequences from corresponding TCR sequence group. Within each group, each sequence set contains 2–10 sequences with randomly assigned weights that sum to 100. 45 string set pairs are generated within each group.

S1.2 Heterogeneous sets of Hepatitis B Virus genomes

We downloaded 30 HBV genomes from each of three host species — human, bat, duck — from the NCBI virus database [1]. We construct 3 string sets for each host species. For each string set, we randomly select 5

*Corresponding Author. Email: carlk@cs.cmu.edu

HBV genomes from one host and randomly assign a weight to each string so that the sum of string weights in each set is equal to 100.

S2 The scalability of FGTED

We compare the running time in real time of computing FGTED between graphs constructed with TCR sequences and HBV genomes (Figure S1). We ran all our experiments on a server with 24 cores (48 threads) of two Intel Xeon E5 2690 471 v3 @ 2.60GHz and 377 GB of memory. The system was running Ubuntu 18.04 with Linux kernel 472 4.15.0.

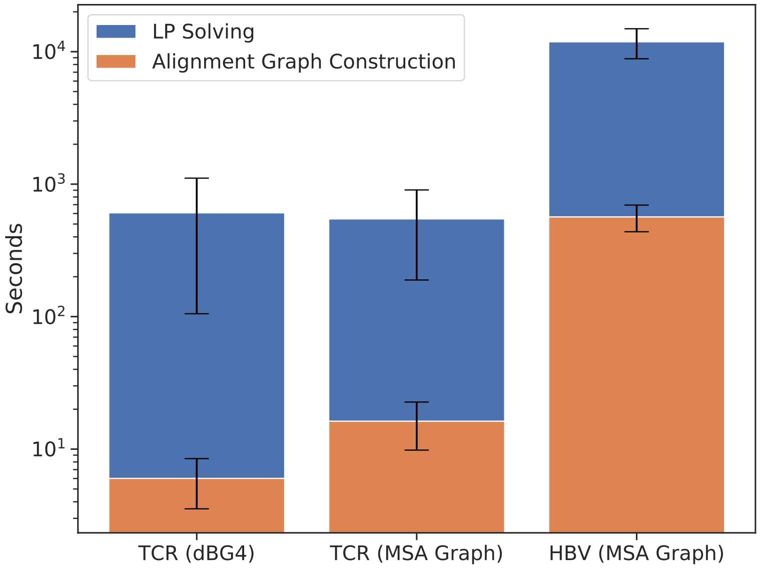

Figure S1: The time taken in the two steps to compute FGTED with dBG4s and MSA graphs. The length of each bar is the average running time for each graph. The error bars represent the standard deviations. Y-axis is in log-scale.

References

[1] Eneida L Hatcher, Sergey A Zhdanov, Yiming Bao, Olga Blinkova, Eric P Nawrocki, Yuri Ostapchuck, Alejandro A Schäffer, and J Rodney Brister. Virus variation resource–improved response to emergent viral outbreaks. *Nucleic Acids Research*, 45(D1):D482–D490, 2017.

[2] Marie-Paule Lefranc and Gérard Lefranc. *The immunoglobulin factsbook*. Academic press, Cambridge, MA, United States, 2001.
